# Circulating long non-coding RNAs as novel diagnostic biomarkers for Alzheimer’s disease (AD): A systematic review and meta-analysis

**DOI:** 10.1371/journal.pone.0281784

**Published:** 2023-03-22

**Authors:** Parnian Shobeiri, Sanam Alilou, Mehran Jaberinezhad, Farshad Zare, Nastaran Karimi, Saba Maleki, Antonio L. Teixeira, George Perry, Nima Rezaei

**Affiliations:** 1 Children’s Medical Center Hospital, School of Medicine, Tehran University of Medical Sciences (TUMS), Tehran, Iran; 2 Network of Immunity in Infection, Malignancy and Autoimmunity (NIIMA), Universal Scientific Education and Research Network (USERN), Tehran, Iran; 3 Research Center for Immunodeficiencies, Pediatrics Center of Excellence, Children’s Medical Center, Tehran University of Medical Sciences, Tehran, Iran; 4 School of Medicine, Iran University of Medical Sciences, Tehran, Iran; 5 Student Research Committee, Tabriz University of Medical Sciences, Tabriz, Iran; 6 Clinical Research Development Unit of Tabriz Valiasr Hospital, Tabriz University of Medical Sciences, Tabriz, Iran; 7 School of Medicine, Sari Branch, Islamic Azad University, Sari, Iran; 8 School of Medicine, Guilan University of Medical Sciences, Rasht, Iran; 9 Neuropsychiatry Program, Department of Psychiatry and Behavioral Sciences, McGovern Medical School, The University of Texas Health Science Center at Houston, Houston, TX, United States of America; 10 Department of Biology and Neurosciences Institute, University of Texas at San Antonio (UTSA), San Antonio, TX, United States of America; 11 Department of Immunology, School of Medicine, Tehran University of Medical Sciences, Tehran, Iran; Shahid Sadoughi University of Medical Sciences and Health Services, ISLAMIC REPUBLIC OF IRAN

## Abstract

**Background:**

Long non-coding RNAs (lncRNAs) have been reported to be involved in the pathogenesis of neurodegenerative diseases. It has also been hypothesized that plasma exosomal lncRNAs may be used as Alzheimer’s disease (AD) biomarkers. In this systematic review, we compiled all studies on the subject to evaluate the accuracy of lncRNAs in identifying AD cases through meta-analysis.

**Methods:**

A PRISMA-compliant systematic search was conducted in PubMed/MEDLINE, EMBASE, and Web of Science databases for English publications till September 2022. We included all observational studies published which investigated the sensitivity and specificity of various lncRNAs in plasma samples of AD diagnosis. Our search strategy included lncRNA and all the related spelling and abbreviation variations combined with the keyword Alzheimer’s disease. Methodological quality was assessed using the Strengthening the Reporting of Observational Studies in Epidemiology (STROBE) guidelines and the Quality Assessment of Diagnostic Accuracy Studies (QUADAS-II) tool. The meta-analysis was carried out using the area under the Receiver Operator Characteristic (ROC) curves (AUC) and sensitivity and specificity values to assess the accuracy of the identified lncRNAs in AD diagnosis. To account for the predicted heterogeneity of the study, a random-effects model was used. All the statistical analyses and visualizations were conducted using Stata 17.0 software.

**Results:**

A total of seven studies (AD patients = 553, healthy controls = 513) were included in the meta-analysis. Three lncRNAs were upregulated (RNA BACE-AS1, RNA NEAT1, RNA GAS5), and one lncRNA (MALAT1) was downregulated in plasma samples of AD patients. RNA 51A and RNA BC200 were reported to have variable expression patterns. A lncRNA (RNA 17A) was not significantly different between AD and control groups. The pooled sensitivity, specificity, and AUC values of lncRNAs in identifying AD were (0.74; 95% CI [0.63, 0.82], I2 = 79.2%), (0.88; 95% CI [0.75, 0.94], I2 = 88.9%), and 0.86; 95% CI [0.82, 0.88], respectively. In addition, the pooled diagnostic odds ratio (DOR) of the five individual lncRNAs in AD diagnosis was 20.

**Conclusion:**

lncRNAs had high accuracy in identifying AD and must be seen as a promising diagnostic biomarker of the disease.

## Introduction

Alzheimer’s disease (AD) is the most common neurodegenerative disease evolving with cognitive decline and dementia [1, 2]. From a pathological perspective, AD is defined by the presence of plaques of amyloid-beta (Aβ) peptides (derived from the proteolytic cleavage of the amyloid precursor protein) and neurofibrillary tangles (produced by hyperphosphorylated tau protein forms) [3]. Currently, there is no disease-modifying therapy capable of reversing AD [4], but there are treatments that may improve cognitive and behavioral symptoms [5, 6].

A definitive AD diagnosis can be made after a pathological brain examination [7]. However, in clinical practice, AD diagnosis is essentially clinical, i.e., based on the identification of a typical pattern of symptoms and exclusion of common disorders (e.g., Parkinson’s disease) [7–9]. Both sensitivity (range 53.0%-100%) and specificity (range 55%-99%) of clinical diagnostic criteria are highly variable [10], and none of the clinical diagnostic criteria are satisfactory for identifying pathologically-defined AD [11]. Different methods have been proposed in this context and aim to improve diagnostic accuracy. In association with clinical information, neuroimaging techniques, including various brain single-photon emission computerized tomography (SPECT) types (sensitivity range 63.0%-100%; specificity range 65.0%-100%) and magnetic resonance imaging (MRI) sequences (sensitivity range 72.8%-85%; specificity range 69.0%-89.0%) have provided better diagnostic accuracy, but there are multiple shortcomings, such as radiation exposure in SPECT [12], high cost [13], limited access [14], challenges of patients with metallic devices, claustrophobic and uncooperative patients [10, 15]. Cerebrospinal fluid (CSF) biomarkers, including T-tau, P-tau, and Aβ1–42, have also been widely studied and incorporated into current diagnostic criteria of AD [10, 16]. The invasive nature of collecting CSF biomarkers through lumbar puncture hinders its more widespread use in clinical settings [10]. Aβ-PET imaging (positron emission tomography) and tau PET tracers have demonstrated promising results but these biomarkers rely on neuroimaging techniques that require high-quality and expensive machinery, making them unfeasible for large-scale examinations [17]. Hence, there is a need for other biomarkers that help improve diagnostic accuracy, also allowing diagnosis in the early stages of AD.

Recent studies have implicated non-coding RNAs, especially microRNAs and long non-coding RNAs (lncRNAs), in the pathophysiology of AD [18]. lncRNAs are RNA sequences that usually consist of more than 200 nucleotides that are not transcribed but can regulate genes at the transcriptional, post-transcriptional, and translational levels [19]. lncRNAs can act as miRNA sponges [20], preventing miRNAs from completing their regulatory function. By highly sensitive methods of RNA analysis (e.g., RT-qPCR, microarray analysis), lncRNAs can be detected in tissues and fluids such as blood and urine [21]. Changes in lncRNA expression have been associated with a variety of diseases, including cancers and neurodegenerative diseases [22]. For instance, microRNA-125b (miR-125b) is identified as a target gene of the lncRNA metastasis-associated lung adenocarcinoma transcript 1 (lnc-MALAT1). Downregulation of lnc-MALAT1 by inducing miR-125b overexpression suppresses cell proliferation, promotes tau phosphorylation and apoptosis, and facilitates inflammation in AD [23]. Another lncRNA implicated in AD is beta-amyloid cleaving enzyme-antisense (BACE1-AS) [22]. Faghihi et al. showed that BACE1-AS lncRNA pairs with the BACE1 mRNA and induces drastic changes in the secondary or tertiary structures of the BACE1 mRNA in both *in vitro* human cells and *in vivo* mouse brains. These events lead to increased BACE1 mRNA stability and translation into a positive feed-forward pathway. Therefore, upregulation of BACE1-AS lncRNA induces BACE1 transcripts and increases Aβ peptide production [24]. In this study, we aimed to systematically review and meta-analyze original investigations that assessed the diagnostic accuracy of plasma levels of lncRNAs in AD patients.

## Materials and methods

### Protocol and registration

This systematic review has adhered to the Preferred Reporting Items for Systematic Reviews and Meta-Analyses (PRISMA) guidelines [25].

### Eligibility criteria

In order to assess the diagnostic accuracy of lncRNAs in AD, we included all original studies, which provided AUC, sensitivity, and specificity values of plasma lncRNAs in the detection of AD. We only included original human studies that were written in the English language. We further excluded non-peer-reviewed and non-English studies, reviews, letters, commentaries, case reports, case series, and articles that did not report or analyze true positives, true negatives, false positives, false negatives, and sensitivity or specificity of lncRNAs in AD detection.

### Information source and search strategies

By using the related keywords, a systematic search was done in Medline (via PubMed interface), EMBASE, and Web of Science for English publications until September 2022 without any time limitations. In order to conduct a systematic search, we used the following search keywords in our selected databases: (Alzheimer) AND (“long untranslated RNA” OR “RNA long non-coding” OR “RNA, long untranslated” OR “large intergenic non-coding” OR “large intergenic non-protein-coding RNA” OR “large intergenic non coding RNA” OR “lnc RNA” OR “lncRNA” OR “LNC RNA” OR “LNCRNA” OR “long ncRNA” OR “long ncRNAs” OR “long non coding RNA” OR “long non protein RNA” OR “long non coding RNA” OR “long untranslated RNA”. Detailed searched strategy is available in S1 Table.

### Data management and selection process

A total of 1,258 Articles obtained from our systematic search were imported to Endnote X20 (Clarivate analysis, Philadelphia). After removing the duplicates, two authors (S.A and M.J) screened studies based on their titles and abstracts and identified all eligible studies. Full texts of the included studies were obtained and reviewed independently according to the inclusion and exclusion criteria. The third researcher (P.S) assessed the probable discrepancies between data extraction files, and any disagreements were resolved by consensus.

### Data collection process and data items

From each included study, we extracted the following information, including title, year of publication, authors’ names, number of included participants, type of the lncRNA, sensitivity, specificity, area under the curve, and 95% confidence interval (CI) of the area under cure if available. The data extraction sheet is available in S1 File.

### Risk of bias assessment

To assess the quality of observational studies, we used “strengthening the reporting of observational studies in epidemiology” (STROBE) [26]. The STROBE checklist is comprised of 22 items and some items have been subcategorized into more items. If the subcategorized item was mentioned in the appropriate section of the article, it received one point; if not, a zero point was given. Some items might not have been applicable to a specific article; hence, the items were not considered. The Completeness of reporting (COR) score for each manuscript was calculated: COR score = ∑1 / [∑ 1 + ∑ 0]. Therefore, we considered >70% as "low-risk", 40–70% as "medium risk", and <40% as "high risk". Two reviewers assessed the risk of bias and quality assessment in the studies included in the meta-analysis utilizing the STROBE and QUADAS-II checklist (www.bris.ac.uk/quadas/quadas-2) [27], respectively. A third researcher was consulted whenever the other two reviewers were unsure how to score an item or the study.

### Statistical approach

The analysis was carried out using the area under the Receiver Operator Characteristic (ROC) curves to assess the accuracy of the identified lncRNAs in identifying the presence of AD, as indicated by the AUC value and, if available, sensitivity and specificity [28]. The meta-analysis included studies that investigated the sensitivity and specificity of various lncRNAs in AD diagnosis. To account for the predicted heterogeneity of research, a random-effects model (DerSimonian-Laird approach) was used [29]. STATA version 17 was used to create all visualizations (StataCorp LP, College, Station, TX, USA). Furthermore, all analyses were carried out using the STATA 17.0 program. The heterogeneity of included studies was examined using I^2^ and χ^2^ statistics, with I^2^ more than 50% or *p-value*<0.05 considered significant.

## Results

### Literature search

**Fig 1:**
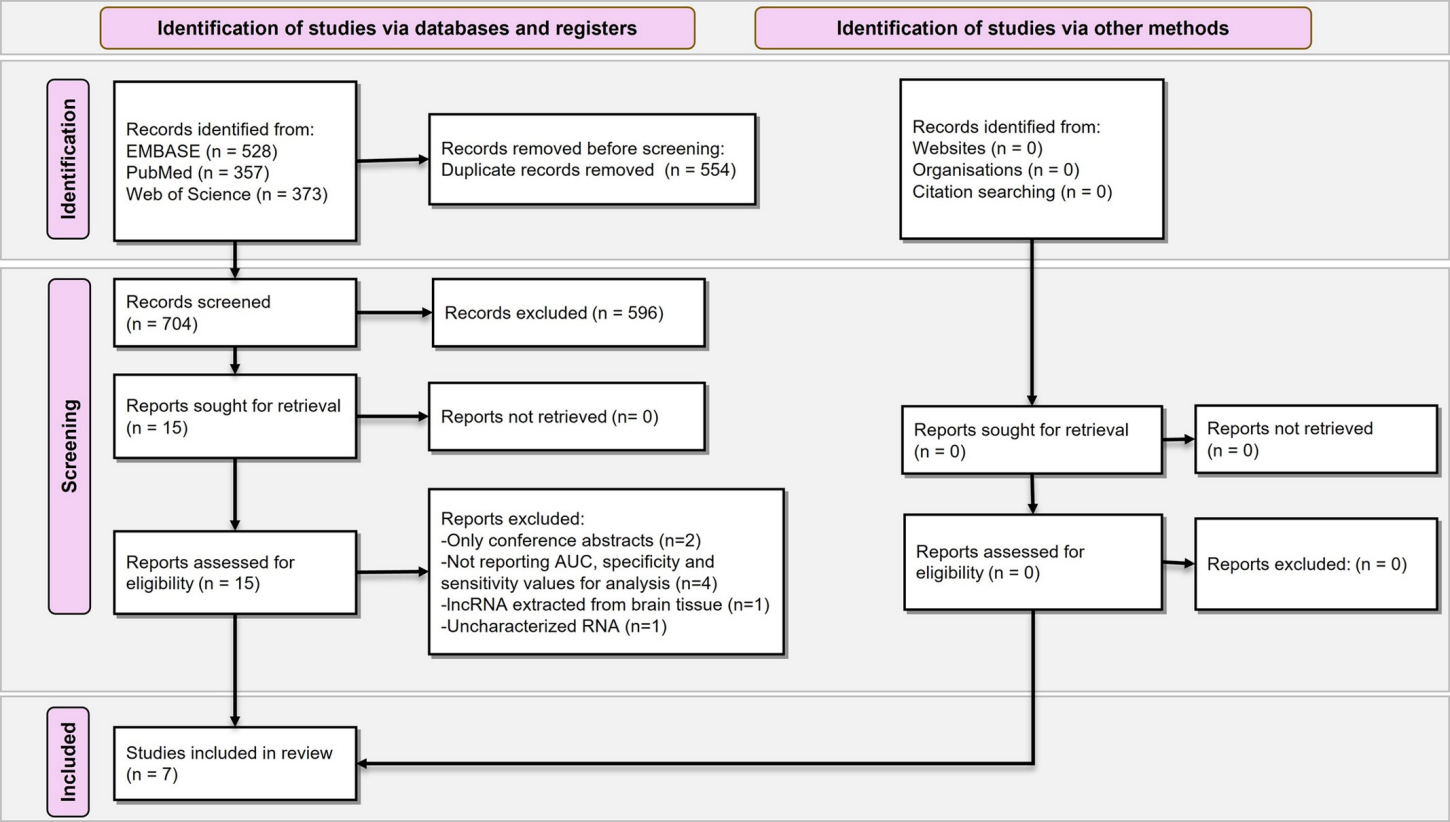
Flow chart of systematic review according to Preferred Reporting Items for Systematic Reviews and Meta-Analysis (PRISMA) guideline.

As presented in the flowchart for study selection in Fig 1, 1,258 records were retrieved from the systematic database search. Five hundred fifty-four duplicate records were automatically removed using EndNote Software, and 704 records were screened. Of them, fifteen records were considered eligible. Moreover, we searched other databases, including ADNI (The Alzheimer’s Disease Neuroimaging Initiative), and we did not find other articles that assessed the relationship between plasma lncRNAs and Alzheimer’s disease. Lastly, seven studies were included in the final meta-analysis. The study selection process is illustrated as a PRISMA flow diagram (Fig 1).

### Study characteristics and quality assessment

The main characteristics of the seven studies included in the systematic review are summarized in Table 1. A total of 602 AD patients and 576 healthy controls from China [23, 30–33] and Iran [22, 34] were included in the review. The main method for detecting lncRNAs in the included studies was Reverse Transcription and Quantitative Real-Time PCR (RT-qPCR). According to the STROBE checklist, all included studies were deemed to have a low risk of bias (Table 2). Overall, there were no applicability concerns in the selected studies. Also, based on the QUADAS-II checklist, all included studies were assessed as having a low risk of bias in all four domains (Table 3).

**Table 1:** Summary of peer-reviewed studies reporting the association and accuracy of lncrnas collected through plasma samples in patients with Alzheimer’s disease.

First author	Year/Country	Study Design	Sample size	Case	Control	lncRNA name	*p*-value	Up/down regulation	Sensitivity (95%CI)	Specificity (95%CI)	AUC (95%CI)
**Feng**	2018/China	Case-Control (diagnostic study)	160	88	72	RNA 17A	0.054	Upregulation	NA	88%	0.629 (0.511–0.747)
						RNA 51A	0.113				0.596 (0.579–0.714)
						RNA BACE1-AS	0.003				0.667 (0.553–0.781)
						RNA BC200	0.123				0.594 (0.477–0.711)
**Wang**	2020/China	Case-Control (diagnostic study)	134	72	62	RNA BACE1-AS	<0.005	Upregulation	87.5%	61.3%	0.761 (0.675–0.848)
						RNA BC200	0.354				0.54 (0.436–0.645)
						RNA 51A	0.451				0.54 (0.436–0.643)
**Fotouhi**	2019/Iran	Case-Control (diagnostic study)	81	45	36	RNA BACE1-AS	<0.001	Upregulation	75%	100%	0.98 (0.81–0.966)
**Zhuang**	2020/China	Case-Control (diagnostic study)	240	120	120	RNA MALAT1	<0.001	Downregulation	NA	NA	0.83 (0.79–0.888)
**Deng**	2017/China	Case-Control (diagnostic study)	160	70	90	RNA 51A	<0.001	Upregulation	83.9%	72.9%	0.844
**Chen**	2022/China	Case-Control (diagnostic study)	191	108	83	RNA GAS5	<0.001	Upregulation	61.1%	95.2%	0.831
**Khodayi**	2022/Iran	Case-Control (diagnostic study)	100	50	50	RNA BC200	0.02	Upregulation	60%	91%	0.79 (0.706–0.886)
						RNA NEAT 1	0.0021	Upregulation	72%	84%	0.85 (0.776–0.924)

AUC: area under cure, CI95%: confidence interval, LNCRNA: long-noncoding RNA

**Table 2:** Quality assessment of the included studies using the STROBE checklist.

Items of STROBE checklist	Title and abstract		Background/rationale	Objectives	Study design	Setting	Participants		Variables	Data sources/ measurement	Bias	Study size	Quantitative variables	Statistical methods					Participants			Descriptive data			Outcome data	Main results			Other analyses	Key results	Limitations	Interpretation	Generalisability	Funding	Average
**Feng**	1	1	1	1	1	1	1	1	1	1	1	0	1	0	1	N/A	0	1	1	N/A	0	1	1	N/A	1	1	1	N/A	1	1	1	1	1	1	86%
**Wang**	1	1	1	1	1	1	1	1	1	1	1	0	1	1	1	N/A	0	1	1	N/A	0	1	1	N/A	1	1	1	N/A	1	1	1	1	1	1	90%
**Fotuhi**	1	1	1	1	1	1	1	1	1	1	0	0	1	1	1	N/A	0	1	1	N/A	0	1	1	N/A	1	1	1	N/A	1	1	0	1	1	1	83%
**Zhuang**	1	1	1	1	1	1	1	1	1	1	1	0	1	1	1	N/A	1	1	1	N/A	0	1	1	N/A	1	1	1	N/A	1	1	1	1	1	1	93%
**Deng**	1	1	1	1	1	1	1	1	1	1	0	0	1	1	1	N/A	1	1	1	N/A	0	1	1	N/A	1	1	1	N/A	1	1	0	1	1	1	86%
**Chen**	1	1	1	1	1	1	1	1	1	1	0	0	1	1	1	N/A	0	1	1	N/A	0	1	1	N/A	1	1	1	N/A	1	1	1	1	1	1	86%
**Khodayi**	1	1	1	1	1	1	1	1	1	1	0	0	1	1	1	N/A	0	1	1	N/A	0	1	1	N/A	1	1	1	N/A	1	1	0	1	1	1	83%

**Table 3:** Quality assessment of the included studies using the QUADAS-II checklist.

	DOMAINS															
Author	Domain 1: Patient Selection				Domain 2: Index Test					Domain 3: Reference Standard				Domain 4: Flow and Timing		
	Risk of Bias (Could the Selection of Patients Have Introduced Bias?)				Applicability concerns	Risk of Bias (Could the Conduct or Interpretation of the Index Test Have Introduced Bias?)			Applicability concerns	Risk of Bias (Could the Reference Standard, Its Conduct, or It’s Interpretation Have Introduced Bias?)			Applicability concerns	Risk of Bias (Could the Patient Flow Have Introduced Bias?)		
	Signaling questions (yes, no, unclear)			Total (High, low, unclear)		Signaling questions (yes, no, unclear)		Total (High, low, unclear)		Signaling questions (yes, no, unclear)		Total (High, low, unclear)		Signaling questions (yes, no, unclear)		Total (High, low, unclear)
	*Was a consecutive or random sample of 30 and more patients enrolled*?	Patients matched with controls or no significant variation between cases and controls	*Did the study avoid inappropriate exclusions*?		*Are There Concerns That the Included Patients and Setting Do Not Match the Review Question*?	Were the index test results interpreted without knowledge of the results of the reference standard?	If a threshold was used, was it pre-specified?		*Are There Concerns That the Index Test*, *Its Conduct*, *or Its Interpretation Differ from the Review Question*?	Is the reference standard likely to correctly classify the target condition?	Were the reference standard results interpreted without knowledge of the results of the index test?		*Are There Concerns That the Target Condition as Defined by the Reference Standard Does Not Match the Question*?	Was there an appropriate interval between index test(s) and reference standard?	*Did all patients receive the reference standard*?	
Feng	Unclear	Yes	Yes	Low	Low	No	Unclear	Low	Low	Yes	Yes	Low	Low	Yes	Yes	Low
Wang	Unclear	Yes	Yes	Low	Low	No	Unclear	Low	Low	Yes	Yes	Low	Low	Yes	Yes	Low
Fotuhi	Unclear	Yes	Yes	Low	Low	No	Unclear	Low	Low	Yes	Yes	Low	Low	Yes	Yes	Low
Zhuang	Yes	Yes	Yes	Low	Low	No	Unclear	Low	Low	Yes	Yes	Low	Low	Yes	Yes	Low
Deng	Unclear	Yes	Yes	Low	Low	No	Unclear	Low	Low	Yes	Yes	Low	Low	Yes	Yes	Low
Chen	Unclear	Unclear	Unclear	Unclear	Low	No	Unclear	Low	Low	Yes	Yes	Low	Low	Yes	Yes	Low
Khodayi	Unclear	Yes	Yes	Low	Low	No	Unclear	Low	Low	Yes	Yes	Low	Low	Yes	Yes	Low

### Significantly dysregulated plasma lncRNAs in AD patients

Among seven lncRNAs, three lncRNAs were reported to be upregulated (RNA BACE-AS1, RNA NEAT1, RNA GAS5) [22, 30, 31, 34]. BACE1-AS lncRNA was confirmed in more than one study as significantly upregulated in plasma samples [22, 31, 32]. One lncRNA (MALAT1) was downregulated in AD patients’ plasma samples [23]. Moreover, RNA 51A and RNA BC200 were reported to have variable expression patterns. Deng et al. and Khodayi et al. reported, respectively, significant upregulation of RNA 51A and BC200 in the plasma of AD patients compared to healthy controls [32, 34]. However, Feng et al. [30] and Wang et al. [31] did not find any significant difference between AD and healthy controls regarding 51A and BC200 lncRNA. Feng et al. also reported that RNA 17A had no significant differences among AD and control groups [30].

### Diagnostic accuracy of circulating lncRNAs in AD patients

**Fig 2:**
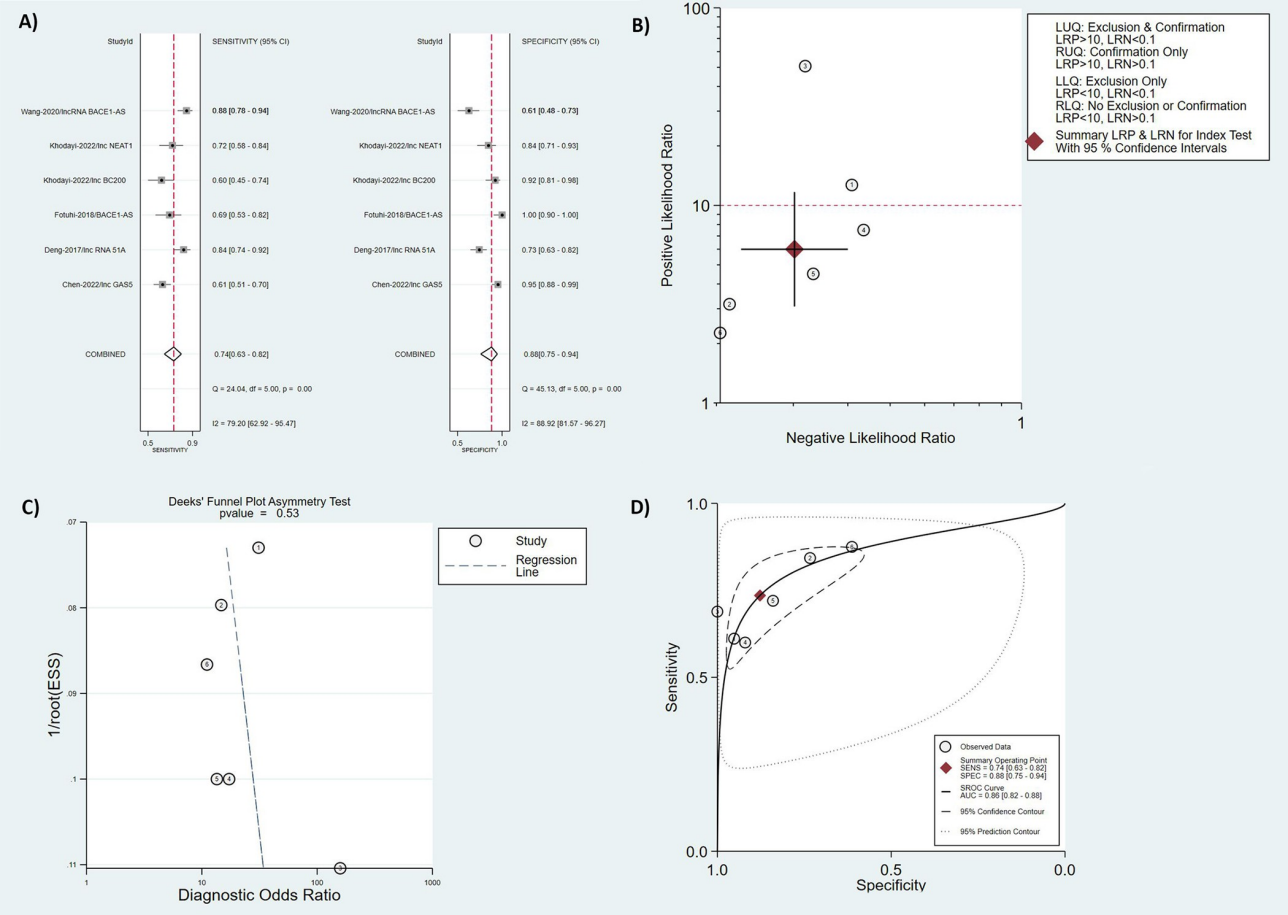
Meta-analysis of diagnostic accuracy of lncRNAs in AD patients vs. HCs (sensitivity and specificity). A: Forest Plot. B: Summary of Positive and Negative Likelihood Ratio. C: Funnel Plot. D: SROC Curve.

Five studies reported sensitivity and specificity values of lncRNAs in diagnosing AD patients [22, 31–34]. Within these five studies, the above-mentioned parameters for five lncRNAs were reported. The pooled sensitivity and specificity values of lncRNAs in diagnosing AD are (0.74; 95% CI [0.63, 0.82], I^2^ = 79.2%), and (0.88; 95% CI [0.75, 0.94], I^2^ = 88.9%), respectively (Fig 2A). Based on these findings, lncRNAs are highly discriminative when used as biomarkers in the detection of AD. Moreover, the values of positive and negative likelihood ratios are (6; 95% CI [3.1, 11.7]), and (0.30; 95% CI [0.23, 0.40]), respectively (Fig 2B). Regarding publication bias, Deek’s funnel plot asymmetry test [35] was not statistically significant (*p-value* = 0.53, Fig 2C), and therefore there is a low possibility of publication bias in the performed meta-analysis. The summary receiver operating characteristic (SROC) has been plotted, and the calculated area under the curve (AUC) was (0.86; 95% CI [0.82, 0.88], Fig 2D). A random-effects model was used to re-analyze the data. The diagnostic threshold was also examined, and Spearman’s correlation coefficient was 1, showing that the diagnostic threshold contributed to significant heterogeneity. The existing heterogeneity could also be attributed to differences in study techniques, specimen types, endogenous references, or sample sizes. In addition, the pooled diagnostic odds ratio (DOR) of the five individual lncRNAs in AD diagnosis was 20.

**Fig 3:**
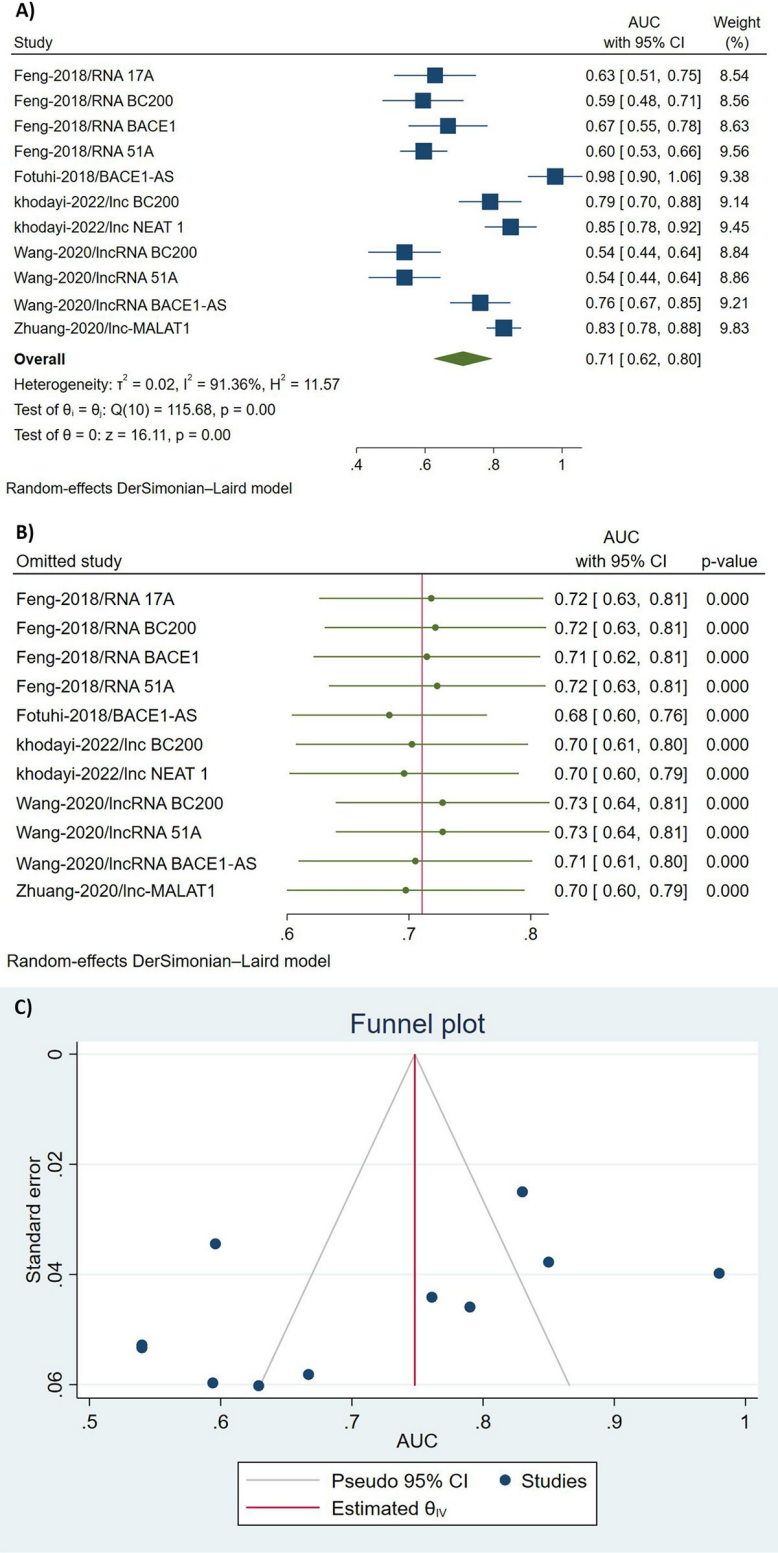
Meta-analysis of diagnostic accuracy of lncRNAs in AD vs. HCs (AUC). A: Forest Plot. B: Leave-one-out analysis. C: Funnel Plot.

Five of the included studies [22, 23, 30, 31, 34] reported the AUC values of lncRNAs in detecting AD, and the pooled AUC value of seven individual lncRNAs were (0.711; 95% CI [0.624; 0.797], I^2^ = 91.3%, Fig 3A). A leave-one-out analysis was performed due to the high heterogeneity to determine which studies contribute to the most heterogeneity. Fig 3B illustrates the results of the leave-one-out analysis. Regarding the publication bias, the Begg’s (*p-value* = 0.27) and Egger’s (*p-value* = 0.051) tests were insignificant. Accordingly, there was no evidence of publication bias, and the funnel plot was symmetric (Fig 3C).

### Diagnostic accuracy of circulating BACE1-AS lncRNA in AD patients

**Fig 4:**
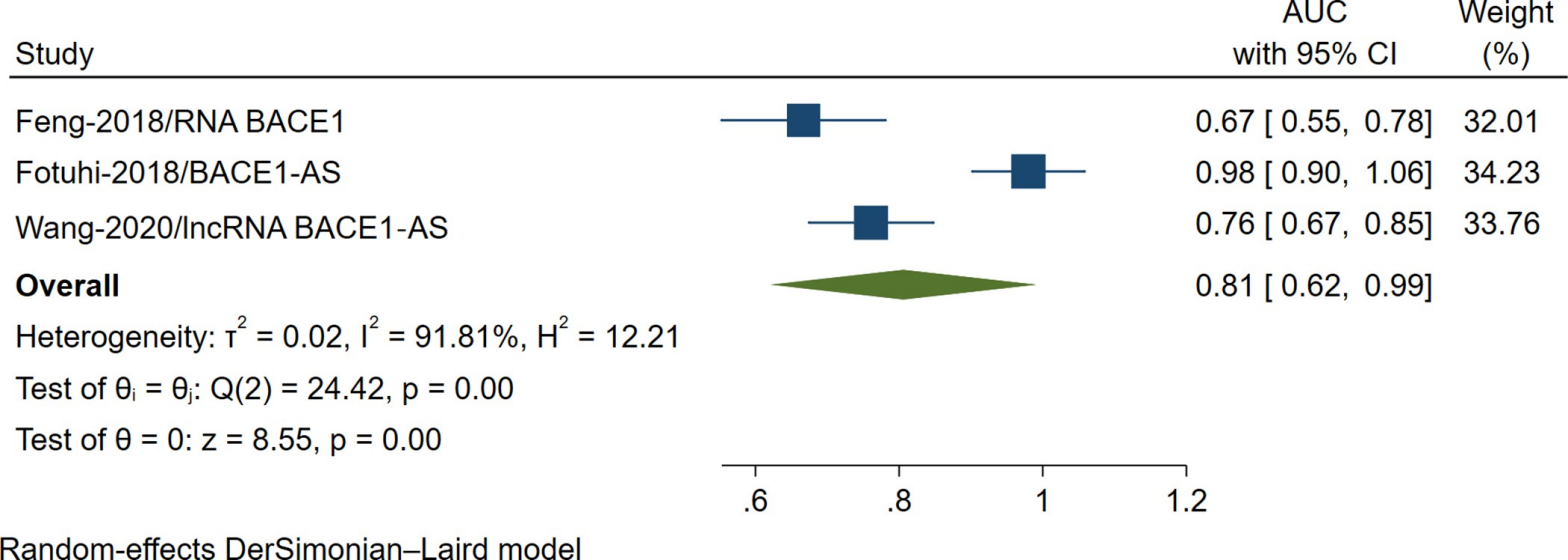
Forest plot of the meta-analysis of diagnostic accuracy of BACE1-AS lncRNA in AD vs. HCs (AUC).

Three studies reported diagnostic accuracy of BACE1-AS lncRNA in diagnosing AD patients, and the pooled AUC value of these three studies were (0.806; 95% CI [0.621; 0.991], I^2^ = 91.81%, Fig 4) [22, 30, 31].

## Discussion

Our study managed to capture a glimpse of lncRNA as a potential biomarker of AD. The present study showed that the plasma lncRNA levels of AD patients have the potential to be useful biomarkers for AD diagnosis. The pooled sensitivity and specificity values of lncRNAs in diagnosing AD were 74% and 88%, respectively.

All three studies that compared BACE1-AS in AD patients and healthy groups showed significant upregulation of this lncRNA [22, 30, 31]. BACE1-AS increases the stability of the BACE1 mRNA by directly interacting with the BACE1 mRNA, resulting in increased levels of the BACE1 protein and increased generation of Aβ1–40 and Aβ1–42 from amyloid precursor protein (APP) through the β secretase pathway. Increased Aβ levels lead to increased expression of BACE1-AS, thus forming a positive feedback loop [24, 36]. The pooled AUC for studies investigating this lncRNA, shows promise as a potential diagnostic tool. Unfortunely not all of them reported sensitivity and specificity. Hence, we could not pool the data on these variables.

**Fig 5:**
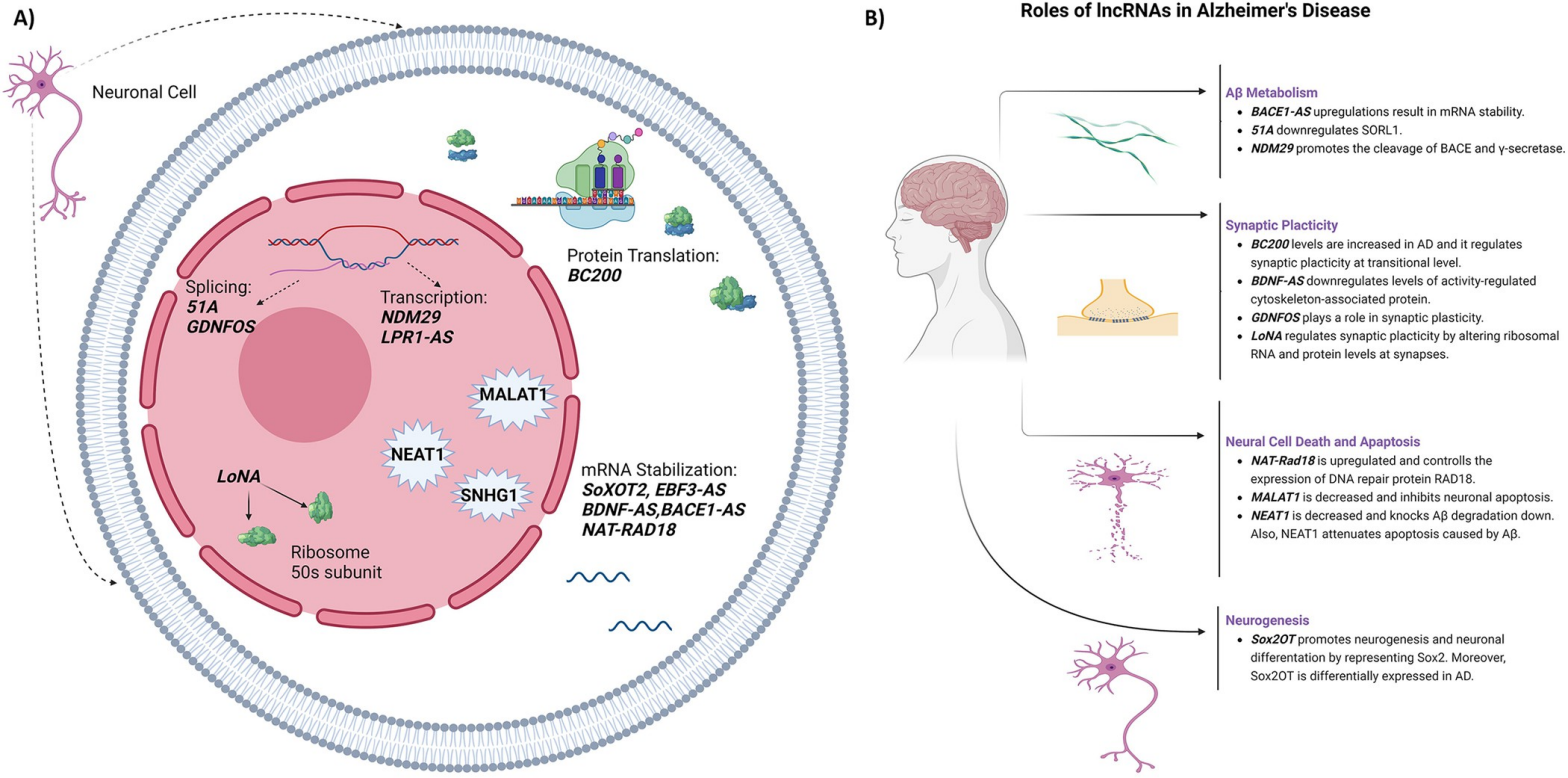
A: The roles of lncRNAs in neuronal cells in the pathogenesis of AD. B: The classification of roles for lncRNAs in AD. (made by biorender.com).

BC200 is a 200-nucleotide predominantly cytoplasmic lncRNA that is selectively targeted to somatodendritic domains of neurons. In AD, plasticity failure has been proposed to be the starting point of neurodegeneration [37]. BC200 RNA is thought to operate as a modulator of local protein synthesis in postsynaptic dendritic microdomains and contribute to maintaining long-term synaptic plasticity [37]. BC200 has shown a seemingly contradicting profile in AD patients. Jukiw et al. demonstrated a 70% reduction in BC200 signal strength in AD brains (Brodman area 22), whereas non-AD neocortices showed a strong presence of BC200. This finding goes in line with the observation that AD brains exhibit marked deficits in the abundance of neuron-specific DNA transcripts [38]. In contrast, Mus et al. observed that BC200 levels in Brodmann’s area 9 were substantially higher than in control brains of comparable age. Moreover, BC200 levels were considerably higher in area 9 than in area 17 (primary visual cortex, a region that is typically spared in AD), and BC200 levels in area 17 of AD cases did not significantly differ from those in area 9 or 17 of controls. Accordingly, overexpression of BC200 RNA may be reactive/compensatory to, or causative of, synaptodendritic deterioration in AD neurons [37]. Other studies compared the level of BC200 RNA in plasma samples of AD and control groups. Feng et al. and Wang et al. showed no significant difference in plasma levels of BC200 RNA between patients and control groups [30, 31]. Feng et al. also showed mild-moderate positive correlations between LncRNA 51A with LncRNA BC200 in AD patients (r = 0.78, p < 0.001) [30]. In contrast with these two studies, Khodayi et al. found that BC200 plasma levels were significantly higher in AD compared with control. BC200 discriminated advanced AD patients (with a Mini-Mental State Examination (MMSE) score of less than 19) and subjects labeled as pre-clinical (with MMSE scores of 24–26) from healthy controls with a sensitivity of 60% and 83% and specificity of 91% and 66%, respectively [34]. It is noteworthy to mention the study of Li et al. that evaluated the role of BC200 on viability and apoptosis of cells overexpressing Aβ1–42. BC200 regulated cell viability and apoptosis via targeting BACE1. Knockdown of BC200 significantly suppressed BACE1 expression, while overexpression of BC200 increased BACE1 expression. Furthermore, inhibition of BC200 significantly increased cell viability and reduced cell apoptosis via directly targeting BACE1 [39] (Fig 5).

A previous study has shown that lncRNA 51A is frequently upregulated in cerebral cortices of AD. 51A expression drives a splicing shift of SORL1 (sortilin-related receptor 1) from the canonical long protein variant A to an alternatively spliced protein form. SORL1 is proposed to be a regulator of endosomal traffic and recycling in human neurons. This process resulted in decreased synthesis of SORL1 variant A, which led to impaired processing of amyloid precursor protein (APP) and increased Aβ formation [40]. Moreover, Deng et al. found that 51A RNA was upregulated in the plasma of AD patients compared with controls, and 51A RNA was negatively correlated with the MMSE score in AD patients [32]. Other studies did not find significant differences between patients and control groups regarding 51A [30, 31].

Considering the potential neuroprotective function and anti-inflammatory effect of lnc-MALAT1 on different neurological diseases, previous studies examined whether lnc-MALAT1 might have a protective role in the pathology of AD [41]. Ma and colleagues found that lnc-MALAT1 overexpression inhibited neuron apoptosis, promoted neurite outgrowth, reduced IL-6 and tumor necrosis factor- α (TNF-α) levels, and increased IL-10 levels compared to control overexpression. Additionally, lnc-MALAT1 targets miR-125 (proved to have an essential gene contributing to AD development and progression by promoting neuron cell apoptosis and increasing tau phosphorylation) and reversely regulates miR-125b expression [41]. In another study, Li and colleagues explored the neuro-protective roles of MALAT1 in AD rat models. They found that MALAT1 could promote neuronal recovery following AD through the miR-30b/CNR1 network and the activation of PI3K/AKT signaling [42]. Zhuang et al. reported that the plasma level of MALAT1 decreased, while miR-125b and PTGS2 were elevated in AD patients compared with Parkinson’s disease and controls. Moreover, plasma MALAT1, miR-125b, FOXQ1, PTGS2, and CDK5 could distinguish AD patients from controls, while only plasma MALAT1, miR-125b, and PTGS2 could discriminate AD patients from Parkinson’s disease patients [23]. This latter finding is important, suggesting that the observed changes might be specific to AD and not related to neurodegenerative processes.

Massone and colleagues reported that upregulation of 17A would impair gamma aminobutyric acid (GABA) signaling, enhance Aβ secretion, and increase the Aβ-42/Aβ-40 ratio [43]. However, Feng et al. did not find a significant difference in the expression of 17A between AD patients and control groups (r = 0.82, p < 0.001) [30].

NEAT1 is highly expressed in the brain of AD patients [34], playing pathological effects in other neurodegenerative diseases such as Huntington’s and Parkinson’s diseases [44]. Ke and colleagues investigated the neuro-regulator function of NEA1 in Aβ-treated SH-SY5Y and SK-N-SH cells (cloned subline of a neuroblastoma cell line). They found that NEAT1 expression was remarkably upregulated in this model. Silencing NEAT1 attenuated Aβ-induced inhibition of cell viability and increased apoptosis and phosphorylation of Tau [44]. Two recent studies confirmed that NEAT1 is highly expressed in AD patients’ temporal cortex and hippocampus [45, 46]. Khodayi et al. [34] reported that NEAT1 could efficiently discriminate between mild cognitive impairment and Advance-AD with 60.73% sensitivity and 91.71% specificity, respectively.

GAS5 is an anti-oncogene extensively studied in tumors [33]. Cao and colleagues found that silencing GAS5 improved functional recovery, suppressed neuron cell apoptosis, and inhibited the inflammatory response in HT22 cells [47]. Chen et al. demonstrated GAS5 was upregulated in patients with AD and correlated with cognition, as assessed by MMSE. These findings suggested that GAS5 may be associated with the progression of AD. Interestingly, the expression of GAS5 was negatively correlated with hippocampal volume, suggesting that GAS5 may play an important regulatory role in hippocampal atrophy [33]. In contrast, Khodayi et al. did not find a significant difference in GAS5-AS expression in plasma cells of AD patients compared with healthy controls [34].

An important point to consider is the cost of these tests once they are commercially available. If the price is prohibitively high, it would be complex to justify their widespread use. Compared to CSF tests that require relatively invasive sampling through a lumbar puncture and costly imaging alternatives like PET and MRI, serum-based tests could be a much more convenient option to support clinical diagnosis or as a screening method [9]. Furthermore, the value of diagnostic tools will be more relevant when and if disease-modifying therapies (DMT) are introduced [48].

## Limitations

In this review, studies assessing the plasma levels of lncRNAs in AD patients versus healthy age-matched adults were summarized. As a result, there is a chance that some of these lncRNA profiles are also altered in other types of neuronal injury and dementia. For example, MALAT1 dysregulation has already been described in Parkinson’s disease [49]. The issue of specificity also involves other conditions. There is a substantial body of research on BC200 in human cancers [50]. Another limitation of our study was the scarcity of research on the subject. Accordingly, we decided to pool data from different lncRNAs, which led to high heterogeneity.

A study of mechanisms of how these lncRNAs affect AD pathophysiology could also be of great value and deepen our knowledge of the disease. Similarly, they could be used as markers of response to treatment. Currently, any study that adds to this knowledge base will require new systematic reviews to incorporate their results—having an online database listing all potentially relevant. Further studies should focus on exploring the lncRNAs that may display a unique profile or signature in AD patients compared to healthy controls and matched samples of people diagnosed with other diseases, especially neurodegenerative ones. Another next step will be investigating these biomarkers in longitudinal studies using serial testing and assessing their efficacy in early diagnosis and tracking disease progression from mild cognitive impairment to different stages of AD. Furthermore, we did not register our systematic review and meta-analysis prospectively, which may be considered as a potential limitation for the current manuscript.

## Conclusion

Assessment of certain lncRNA levels in plasma seems promising as diagnostic biomarkers of Alzheimer’s disease.

## Supporting information

S1 File Data extraction sheet. (XLSX)

S1 Table Search strategy for each database. (DOCX)

S2 Table PRISMA 2020 checklist. (DOCX)
