# Supplementary material for: Circulating long non-coding RNAs as novel diagnostic biomarkers for Alzheimer’s disease (AD): A systematic review and meta-analysis
Source: PLoS One. 2023 Mar 22;18(3):e0281784. doi: 10.1371/journal.pone.0281784 (PMC10032479; doi:10.1371/journal.pone.0281784)
Supplement: S1 Table — (DOCX) [file pone.0281784.s002.docx]

***Supplementary Material-Search Strategy***

**Circulating long non-coding RNAs as novel diagnostic biomarkers for Alzheimer’s Disease (AD): A systematic review and meta-analysis**

Parnian Shobeiri, Sanam Alilou, Mehran Jaberinezhad, Farshad Zare, Nastaran Karimi, Saba Maleki, Antonio L. Teixeira, George Perry, Nima Rezaei

***Supplementary Table 1.*** *Search strategy for each database*

| **Query** | | **Results** |
| --- | --- | --- |
| ***PubMed*** | | |
| #1 | (('long untranslated rna'/exp OR 'rna, long noncoding' OR 'rna, long untranslated' OR 'large intergenic non coding rna' OR 'large intergenic non protein coding rna' OR 'large intergenic noncoding rna' OR 'linc rna' OR 'lincrna' OR 'lnc rna' OR 'lncrna' OR 'long ncrna' OR 'long ncrnas' OR 'long non coding rna' OR 'long non protein coding rna' OR 'long noncoding rna' OR 'long untranslated rna') ) AND (alzheimer*) | **357** |
| ***Embase*** | | |
| #2 | ('long untranslated rna'/exp OR 'rna, long noncoding' OR 'rna, long untranslated' OR 'large intergenic non coding rna' OR 'large intergenic non protein coding rna' OR 'large intergenic noncoding rna' OR 'linc rna' OR 'lincrna' OR 'lnc rna' OR 'lncrna' OR 'long ncrna' OR 'long ncrnas' OR 'long non coding rna' OR 'long non protein coding rna' OR 'long noncoding rna' OR 'long untranslated rna') AND ('alzheimer disease'/exp OR 'alzeimer disease' OR 'alzeimer`s disease' OR 'alzeimers disease' OR 'alzheimer dementia' OR 'alzheimer disease' OR 'alzheimers disease' OR 'alzheimer fibrillary change' OR 'alzheimer fibrillary lesion' OR 'alzheimer neurofibrillary change' OR 'alzheimer neurofibrillary degeneration' OR 'alzheimer neuron degeneration' OR 'alzheimer perusini disease' OR 'alzheimer sclerosis' OR 'alzheimer syndrome' OR 'alzheimer`s disease' OR 'cortical sclerosis, diffuse' OR 'dementia, alzheimer' OR 'diffuse cortical sclerosis' OR 'late onset alzheimer disease') | **528** |
| ***Web of Science*** | | |
| #3 | TOPIC: (alzheimer) AND TOPIC: ('long untranslated rna'/exp OR 'rna, long noncoding' OR 'rna, long untranslated' OR 'large intergenic non coding rna' OR 'large intergenic non protein coding rna' OR 'large intergenic noncoding rna' OR 'linc rna' OR 'lincrna' OR 'lnc rna' OR 'lncrna' OR 'long ncrna' OR 'long ncrnas' OR 'long non coding rna' OR 'long non protein coding rna' OR 'long noncoding rna' OR 'long untranslated rna') | **373** |
